# A Novel High Throughput Assay for Anthelmintic Drug Screening and Resistance Diagnosis by Real-Time Monitoring of Parasite Motility

**DOI:** 10.1371/journal.pntd.0000885

**Published:** 2010-11-16

**Authors:** Michael J. Smout, Andrew C. Kotze, James S. McCarthy, Alex Loukas

**Affiliations:** 1 James Cook University, Cairns, Australia; 2 CSIRO Livestock Industries, Brisbane, Australia; 3 Queensland Institute of Medical Research, Brisbane, Australia; Swiss Tropical Institute, Switzerland

## Abstract

**Background:**

Helminth parasites cause untold morbidity and mortality to billions of people and livestock. Anthelmintic drugs are available but resistance is a problem in livestock parasites, and is a looming threat for human helminths. Testing the efficacy of available anthelmintic drugs and development of new drugs is hindered by the lack of objective high-throughput screening methods. Currently, drug effect is assessed by observing motility or development of parasites using laborious, subjective, low-throughput methods.

**Methodology/Principal Findings:**

Here we describe a novel application for a real-time cell monitoring device (xCELLigence) that can simply and objectively assess anthelmintic effects by measuring parasite motility in real time in a fully automated high-throughput fashion. We quantitatively assessed motility and determined real time IC_50_ values of different anthelmintic drugs against several developmental stages of major helminth pathogens of humans and livestock, including larval *Haemonchus contortus* and *Strongyloides ratti*, and adult hookworms and blood flukes. The assay enabled quantification of the onset of egg hatching in real time, and the impact of drugs on hatch rate, as well as discriminating between the effects of drugs on motility of drug-susceptible and –resistant isolates of *H. contortus*.

**Conclusions/Significance:**

Our findings indicate that this technique will be suitable for discovery and development of new anthelmintic drugs as well as for detection of phenotypic resistance to existing drugs for the majority of helminths and other pathogens where motility is a measure of pathogen viability. The method is also amenable to use for other purposes where motility is assessed, such as gene silencing or antibody-mediated killing.

## Author Summary

Parasitic worms cause untold morbidity and mortality on billions of people and livestock. Drugs are available but resistance is problematic in livestock parasites and is a looming threat for human helminths. Currently, new drug discovery and resistance monitoring is hindered as drug efficacy is assessed by observing motility or development of parasites using laborious, subjective, low-throughput methods evaluated by eye using microscopy. Here we describe a novel application for a cell monitoring device (xCELLigence) that can simply and objectively assess real time anti-parasite efficacy of drugs on eggs, larvae and adults in a fully automated, label-free, high-throughput fashion. This technique overcomes the current low-throughput bottleneck in anthelmintic drug development and resistance detection pipelines. The widespread use of this device to screen for new therapeutics or emerging drug resistance will be an invaluable asset in the fight against human, animal and plant parasitic helminths and other pathogens that plague our planet.

## Introduction

Billions of people are infected with helminths in developing countries, resulting in many thousands of deaths annually [1], [2]. Helminths also plague livestock in developing and developed countries alike, with the global anthelmintic market for livestock and companion animals valued at $US 3.7 billion in 2002 [3]. While chemotherapy is available for most parasitic helminths, widespread use of anthelmintics in livestock has resulted in the emergence of drug-resistant parasites [4], [5]. Mass drug administration campaigns to control human helminth infections are becoming more widespread and early data are emerging indicating the possible emergence of anthelmintic resistance, for example in river blindness caused by *Onchocerca volvulus* where ivermectin has been widely used, as well as in hookworm and schistosome infections [6]–[10].

Despite the impact of helminths on the health of humans and livestock, the anthelmintic pharmacopoeia is small. This is due in part to the high cost and limited financial return from drug development, particularly for human helminth infections. Another, often overlooked impediment to drug development is the lack of objective high throughput screening methods for assessing drug effectiveness [7], [11], [12]. The current gold standard for measuring drug effectiveness for most adult and larval helminth parasites is *in vitro* assessment of worm motility, as measured visually via microscopy and larval development assays for some larval stages. Such an approach is laborious, subjective and difficult to standardize [8], [11]. For example, the cost and effort to standardise testing for larval anthelmintic resistance against four intestinal parasites of livestock across Europe was substantial [13]. In the 1980s an automated screen was developed, the micromotility meter [14], [15]. The unit utilized light disruption to determine helminth movement. While successful in monitoring motility in both larval and adult stages of a range of parasites, the inherent limitations restricted its use to small scale studies [16].

Many research programs are underway to explore the genetic basis of anthelmintic resistance in order to develop molecular diagnostic assays for anthelmintic resistance. However, with the exception of the benzimidazole class of drugs [17], [18], the molecular basis of anthelmintic resistance is poorly understood, precluding development of widely applicable molecular diagnostics at the present time. Assays based on changes in egg output after drug treatment, the so-called fecal egg count reduction test (FECRT), are useful only after resistance has become commonplace in the population (at least 25%). This method, however, is confounded by density-dependent fecundity effects [19]. Furthermore, for some parasites eggs are not easily collected in quantity, or the only developmental stage present in feces is the larval stage (eg. *Strongyloides* sp.).

Assays have been developed in recent years that score worm migration, feeding and development [8], [12], [20], [21]. While these approaches remove some of the subjectivity, they still require visual scoring by skilled operators, precluding the scale up that would for example be required for a drug discovery program. There are regular pleas in the peer-reviewed literature for high-throughput screening methods to facilitate drug development, and to detect emerging resistance [11], [22]–[24]. Indeed, the Tropical Diseases Research network (TDR) of the WHO (http://apps.who.int/tdr/) has developed an international resistance screening network, but due to the limitations of available techniques, the screening methods utilized have remained low- to medium-throughput [8], [25]. And without a scalable, automated, objective assay for helminth viability, drug development and monitoring for drug resistance for neglected tropical diseases will be difficult [26].

## Methods

### Ethics statement

All animals used were maintained in accordance with the guidelines of the Animal Ethics Committee (AEC) of the Queensland Institute of Medical Research (QIMR) and James Cook University, or under the guidelines set out by the F.D McMaster Animal Ethics Committee, CSIRO Livestock Industries. All studies and procedures were reviewed and approved by the Animal Ethics Committees of QIMR or CSIRO (Animal ethics approval number 09/16).

### Preparation of *Haemonchus* L_3_ and eggs

Feces were collected from *H. contortus* infected sheep that were housed at the McMaster Laboratory, CSIRO Livestock Industries, Armidale, New South Wales (NSW), Australia, and then sent by overnight courier to the CSIRO laboratory in Brisbane, Queensland. The nematode isolates were as follows; [1] Kirby 1981 - isolated from the field at the University of New England Kirby Research Farm in Northern NSW in 1981 - these parasites are susceptible to ivermectin (IVM) and levamisole (LEVA) and thiabendazole (TBZ) [27]; [2] Wallangra 2003 - isolated from the Wallangra region of NSW [28] and resistant to LEVA, benzimidazoles, closantel and macrocyclic lactones. To ensure the resistance status of these parasites, sheep harbouring infections were treated with the recommended dose of a macrocyclic lactone 5 weeks after infection; [3] LAWES – an isolate from South East Queensland that is resistant to LEVA and benzimidazoles (including TBZ) [29]. To ensure the resistance status of these parasites, sheep harbouring infections were treated with the recommended dose of LEVA 5 weeks after infection. Nematode eggs were isolated from feces by filtration and sucrose density gradient centrifugation as previously described [30], while L_3_ were collected as they migrated from fecal cultures. For real time cell assay (RTCA) experiments, 3,000 L_3_ were cultured per well of an E-plate (Roche Inc.) in 200 µl of 0.5× PBS (25 mM sodium phosphate pH 7.2, 70 mM NaCl) at 27°C.

### Preparation of *Strongyloides ratti* L_3_

*Strongyloides ratti* L_3_ were obtained as described elsewhere [20]. For RTCA, 300 L_3_ were cultured per well of an E-plate in 200 µl of 0.5× PBS at 21°C.

### Preparation of adult hookworms

Adults of the canine hookworm, *Ancylostoma caninum* were collected from euthanized stray dogs and cultured *in vitro* at 37°C with 5% CO_2_ as described elsewhere [31] with a modification entailing the supplementation of 200 µl of medium per well with 10% fetal calf serum (Invitrogen). For RTCA, culturing was performed using a single adult worm per well of an E-plate. Immobile worms used for dead background controls were determined by visual inspection.

### Preparation of adult *Schistosoma mansoni*

Adult *Schistosoma mansoni* pairs were collected from the mesenteric veins of mice by perfusion in PBS and then transferred to defined culture medium and cultured at 37°C with 5% CO_2_ as described elsewhere [32]. For RTCA, culturing was performed using one pair in 200 µl (one coupled male and female worm) per well of an E-plate. Immobile worms used for dead background controls were determined by visual inspection.

### Automated assessment of helminth motility and egg hatching in real time using RTCA

The motility of all helminth species and developmental stages was assessed using an xCELLigence system (Roche Inc.) that monitors cellular events in real time without the incorporation of labels by measuring electrical impedance across interdigitated micro-electrodes integrated on the bottom of tissue culture E-Plates (see http://www.roche-applied-science.com/sis/xCELLigence/ezhome.html). For all experiments the inter-well spaces of the E-plate were filled with PBS to reduce evaporation. The RTCA controller software (Roche Inc.) was used to determine how the information was gathered from the single plate RTCA unit (Roche Inc.). The first step consisted of a background reading followed by regular user defined reads at 15 sec intervals for adult and L_3_ stages of all helminths tested (now referred to as “worm tests”) and 25 min intervals for *H. contortus* eggs (now referred to as “egg tests”). For worm tests, helminths were cultured in 180 µl of their respective media per well of the E-plate and motility was monitored overnight to obtain a baseline motility reading prior to addition of 20 µl of a 10× solution of each anthelmintic drug. After addition of drugs (see below), helminths were monitored for a further 3–5 days. For egg tests, E-Plate wells were first filled with 230 µl 0.5× PBS. Then a 96 well Multiscreen mesh filter plate (20 µm pore size, Millipore) was aligned on top of the E-plate and filled with 200 µL of 0.5× PBS containing 3,000 eggs. Dilutions of TBZ (see below) were generated so that 100 µl of drug was added to 100 µl of eggs. Culture was undertaken for 48 hours at 27°C with a small fluorescent light placed 60 cm above the plate to encourage egg hatching.

### Addition of anthelmintic drugs

Drugs used were prepared as stock solutions in DMSO at the following concentrations: 5 mg/ml TBZ; 10 mg/ml IVM; 10 mg/ml LEVA, 5 mg/ml praziquantel (PZQ). Drugs were diluted to 10× stocks in the respective tissue culture media for culturing of each parasite and pre-equilibrated for 1 h before addition of 20 µl of 10× drug to 180 µl of media containing helminths as described above. Final working concentrations of drugs were as follows: PZQ for schistosomes −1.6 µg/ml and two-fold serial dilutions from 400–50 ng/ml; TBZ for adult hookworms (100, 20, 10 and 1 µg/ml) and *H. contortus* eggs (three-fold dilutions from 9 µg/ml–0.037 µg/ml); IVM for *H. contortus* L_3_ (three-fold dilutions from 30–0.4 µg/ml) and *S. ratti* L_3_ (two-fold serial dilutions from 2–0.02 µg/ml); LEVA for *H. contortus* L_3_ resistant and sensitive strains – two-fold serial dilutions from 50–0.4 µg/ml. Control worms were cultured in the presence of DMSO equivalent to that used for the highest drug concentration; this group was used to determine 100% motility.

### Determination of IC_50_ values for anthelmintic drugs

Motility index was used to determine IC_50_ values of drugs for adult and L_3_ stages of the helminths tested, and was calculated as the standard deviation (SD) over 800 data points (i.e. 4 readings per min for 200 min) of the cell index (CI) difference from the rolling average over 20 data points (10 proceeding and preceding CI values- 5 min total). One hundred percent motility was determined from the average motility index of the untreated wells, while 0% motility was determined as an average of when the lowest readings flatten out. The motility index averaged over 100 data points (25 min) was converted to percent motility and this figure was used in Graphpad prism 5.0 to calculate and compare IC_50_ values. We used a log (drug concentration) vs normalised response (100%–0%) formula with variable slope and automatic removal of outliers (with default ROUT coefficient used: Q = 1.0%). For analyses where there were insufficient samples for a complete drug dilution series (*Haemonchus* L_3_ vs IVM and hookworm) a standard hill slope (-1) was used with the previously described non-linear analysis. Determination of IC_50_ values for TBZ with *H. contortus* eggs utilized the raw cell index values that were converted to percent hatching from an average of 100% hatching (no drug) and 0% hatching (9 µg/ml TBZ). All other analyses were as stated above for adult worm and L_3_ stages.

### Statistics

Statistical analyses were undertaken using Graphprism 5.0. When data were sufficient to use the variable slope analysis (all except hookworm and *H. contortus* L_3_ vs IVM) the Hill Slope and the LogIC_50_ value were together compared for significant differences using an extra sum-of squares F-test. Hookworm and *H. contortus* L_3_ vs IVM were analysed with a set Hill Slope value of -1 (described above) and subsequently only the LogIC_50_ was compared with the F-test.

## Results

### Cell Index readout

**Figure 1:**
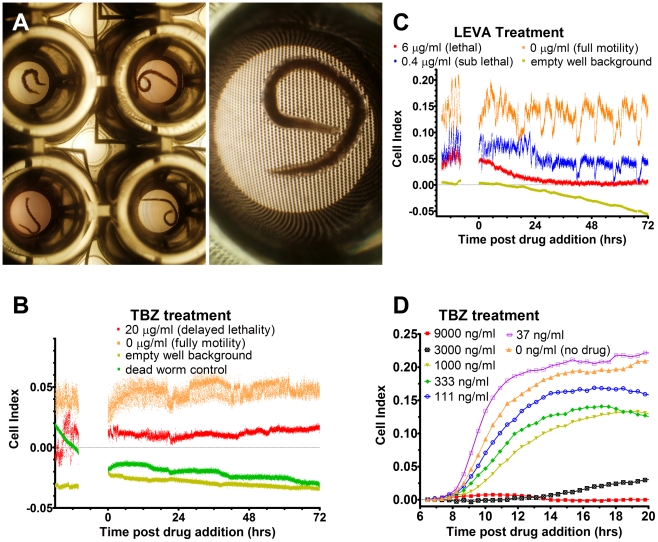
RTCA unit differentiates between live and dead parasites from different developmental stages using the cell index readout. Panel A: Micrograph of adult *Ancylostoma caninum* hookworms - females in the top two wells and magnified image on the left, and males in the bottom two wells. Note the gold circular electrodes covering the base of the E-Plate in the magnified image. Panel B: Cell index output generated by a single adult female *A. caninum* with and without exposure to thiabendazole (TBZ)*. Panel C: *Haemonchus contortus* L_3_ cell index output in the presence of varying amounts of levamisole (LEVA)*. Panel D: *H. contortus* egg hatching in the presence of varying amounts of TBZ - curves show the average of duplicate experiments. Note that increasing drug concentrations result in less egg hatching and a corresponding lesser cell index output. * The cell index numerical value is not relevant to this analysis - the curves have been manually repositioned to assist with visualization of the data. The amplitude within each curve is the important feature of the data for this experiment.

The Real Time Cell Assay (RTCA) unit can differentiate between live and dead parasites at multiple developmental stages for a range of different helminths (Figure 1). The gold electrodes embedded in the base of the wells (Figure 1A) monitor electrical resistance and generate an output presented as a cell index. Larval and adult helminth developmental stages were monitored every 15 sec and the resulting amplitude of the cell index output was proportional to the motility (visual) of the worms (Figures 1B and C). When eggs were monitored using a modified version of the larval migration assay (without the agar overlay) [12], [20], [33] the cell index output was for the most part proportional to the number of hatched larvae that crawled through the nylon mesh and came into contact with the electrodes covering the base of the E-plate (Figure 1D).

### Motility Index and IC_50_

**Figure 2:**
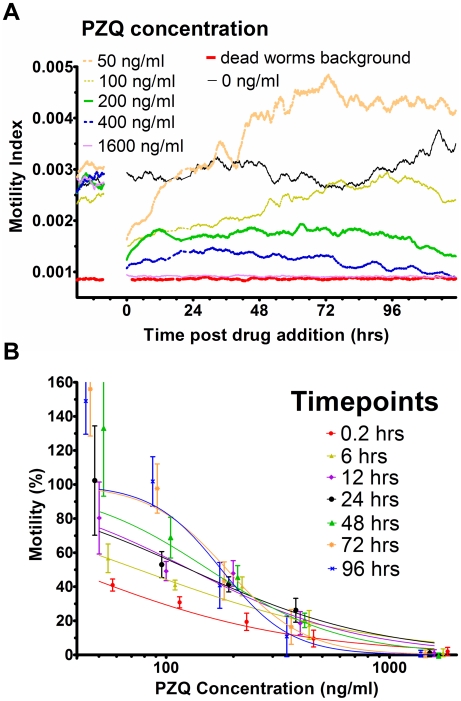
Motility Index of *Schistosoma mansoni* paired adult worms generated from the cell index output. The Motility Index is relative to the amplitude of the cell index curve. Panel A: Drug dilution series with praziquantel (PZQ); each curve is an average of minimum 3 experiments, error bars not shown to enhance clarity. Panel B: PZQ dose response curves used to generate IC_50_ values generated from Motility Index analysis.

**Figure 3:**
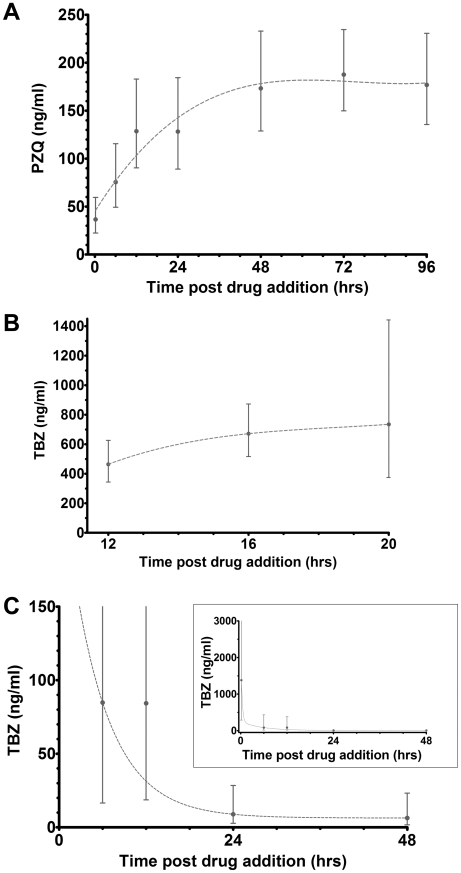
Real time IC_50_ curves with 95% confidence interval error bars for a range of developmental stages of different helminths. Panel A: *Schistosoma mansoni* paired adult worms with praziquantel (PZQ). Panel B: Adult female *Ancylostoma caninum* hookworms with TBZ magnified to aid visualisation; inset shows the entire data set. Panel C: *Haemonchus contortus* eggs with TBZ.

For generation of IC_50_ values the cell index output was converted to a motility index (Figure 2A) which is a measure of the amplitude of the curve scatter. The optimal combination for helminth species and developmental stage was determined as the standard deviation (SD) over 800 data points of the cell index (CI) difference from the rolling average (over 20 data points). The motility index was subsequently converted to a percentage of maximum motility to generate a dose response curve for traditional IC_50_ calculations (Figure 2B). As data is continually monitored, any time point can be selected for IC_50_ analysis. To visualise the effects over time, numerous time points were selected for IC_50_ calculations (Figure 3). As evident from Figure 3, each different helminth and developmental stage exhibited different responses to the drugs tested. For example, the IC_50_ of praziquantel (PZQ) for paired adult schistosomes increased over time and stabilised at 48 hrs (Figure 3A). This is in contrast to the response of female adult hookworms to thiabendazole (TBZ) where the IC_50_ decreased over time but then stabilised at 24 h (Figure 3B), and the response of *H. contortus* egg hatching to TBZ which did not significantly change (Figure 3C).

### Use of RTCA for assessing drug resistance

**Figure 4:**
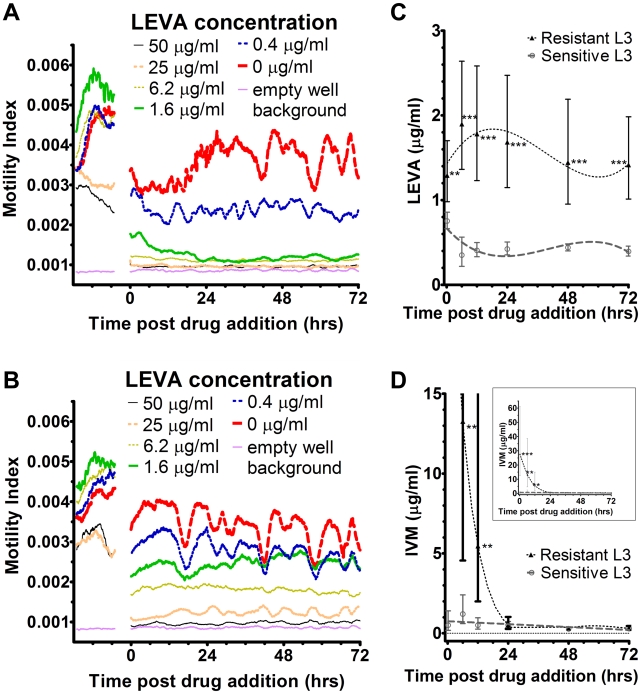
IC_50_ values from RTCA unit can differentiate between LEVA-resistant and -sensitive lines of *H. contortus* L_3_. Panels A and B: Motility Index with selected LEVA concentrations, resistant and sensitive lines respectively. Curves are means of triplicate experiments. Error bars not shown for clarity of the figure. Panel C: Real time IC_50_ curves of LEVA-resistant and -sensitive lines with 95% confidence interval error bars. Panel D: Real time IC_50_ curves of IVM-resistant and -sensitive lines with 95% confidence interval error bars magnified to aid visualisation; inset shows the entire data set. * P<0.05, ** P<0.01, *** P<0.001.

The motility index analysis clearly differentiates between resistant and sensitive strains of *H. contortus* (Figure 4A and B). The IC_50_ values over time (Figure 4C) further demonstrate the differences between motility in levamisole (LEVA) -resistant versus -sensitive lines of *H. contortus* L_3_. Twelve minutes after adding the drug significant (P<0.01) differences were detected between motility of sensitive and resistant lines, and from 6 hours onwards the difference was highly significant (P<0.0001). The curves displaying the IC_50_ over time demonstrated that the LEVA-resistant strain became less motile in a consistent manner, while the motility of the LEVA-sensitive strain decreased after the first reading and then remained steady. The technique also allowed clear differentiation between ivermectin (IVM)-resistant and -sensitive *H. contortus* L_3_ (Figure 4D), where the curves displayed different trends over time. Significant differences in the IVM IC_50_ values between sensitive and resistant lines were apparent over the first 12 hour period but thereafter lost significance.

The data generated from the RTCA unit is summarised and compared to previously published drug sensitivity data in Table 1. In each case the IC_50_ values for the RTCA were lower than those obtained by standard worm motility or egg hatch assays. The differences ranged from 4-fold up to 50-fold.

**Table 1:** Summary of IC_50_ values for a range of drugs and developmental stages of parasitic helminths as measured by RTCA.

Parasite	drug	IC_50_ (ng/ml)	95% CI (ng/ml)	Time to stable IC_50_	Previous data (95% CI) (ng/ml)
female *A. caninum* #	TBZ	13.4	6.3–28.5	6 hrs	unknown
*S. mansoni* adult pairs	PZQ	188	161–221	48 hrs	Varies depending on methodology
*S. ratti* L_3_	IVM	174	103–296	4 hrs	1032 (946–1204) [20]
*H. contortus* L_3_ IVM resistant strain	IVM	310	240–390	24 hrs ^	2950 (1910–4550) (pers. comm. Andrew Kotze 2010)
IVM sensitive strain		280	230–330		1040 (860–1250) [12]
LEVA resistant strain	LEVA	1710	1480–1990	6 hrs **	24 000 (1810–3200) (pers. comm. Andrew Kotze 2010)
LEVA sensitive strain		410	380–440		20 000 (1890–2110) (pers. comm. Andrew Kotze 2010)
*H. contortus* eggs (TBZ resistant strain)	TBZ	704	525–946	12 hrs	4400 (3980–4870) [66]

# Male hookworms were successfully tested but too few worms were available to calculate IC_50_ values.

∧ IVM resistant strain does not show significant IC_50_ difference after stabilisation (24 hrs), but does show a significant difference up to 12 hrs (minimum P<0.01).

**Significantly different to sensitive strain at all time points (minimum P<0.01).

## Discussion

The RTCA unit was developed for automated monitoring of cell growth, from rapid responses over a few minutes to long term studies over a period of weeks [34], [35]. With the ability to monitor adherent cells in a label-free fashion in real time, datasets containing substantially more information than previously obtainable are now being generated. While the system can measure growth of cells in suspension, it requires many more cells than it does for adherent cultures due to the requirement for contact with the electrodes in the bottom of the wells to generate a signal. In fact, any change in the conductivity across the gold electrodes, such as contact, will result in a change in the cell index reading. Live helminth parasites writhe in culture (as they do *in vivo*), and constantly come into contact with the electrodes on the E-plate surface, making the RTCA system ideal for monitoring helminth motility for high-throughput studies. The initial purchase price of the unit might prove an impediment for some laboratories, but the wide ranging of cell based applications and the associated e reduction in manual labour to conduct medium- to high-throughput required will make the system an attractive proposition in the future. Additionally, once the initial RTCA unit and E-plates are purchased, the costs are no greater than those for conventional assays that are currently used for manual monitoring of parasite motility, as the plates are durable and readily reusable. After parasites have been killed by freezing the plates, they can be easily rinsed, sterilized with ethanol and reused many times with minimal reduction in sensitivity and less than 0.2% well failure (data not shown).

Because the RTCA system measures changes in worm motility with a high level of precision, it is widely applicable to a range of helminth species and developmental stages. While we have only tested this technique for the species tested herein (Table 1), it is highly likely that any motile developmental stage from any species that will rest at the bottom of a 96 well microtiter plate can be monitored using minor adaptations of the techniques that we describe here. The ability to directly assess multiple developmental stages for susceptibility to a drug or other intervention is a distinct advantage. For example, PZQ is much more effective against the adult stage of *S. mansoni* than it is against the schistosomulum, the developmental stage that is usually the focus of *in vitro* drug assessments [8]. *H. contortus* displays drug susceptibility differences between infective larval and adult stages, which poses a problem for drug screening and resistance detection that can be overcome by utilizing the RTCA assay for assessing motility of adult worms [36], [37].

Defined skills and experience are generally required to assess worm motility by visual scoring using microscopy. The automated motility index method described herein lends itself to consistency and reproducibility between experiments, between researchers and between laboratories [38]–[40], and thus obviate the requirement for challenging quality assurance programs [13]. The objective nature of the testing removes the subjectivity that afflicts that the majority of current testing methods.

The IC_50_ values obtained by the RTCA were in all cases lower than those obtained from standard motility and egg hatch assays. This is most likely a reflection of the greater sensitivity of the RTCA unit in being able to detect subtle changes in motility that would be missed by the standard methods. The relative ability to detect resistance was mixed - the RTCA more readily detected LEVA resistance than a standard motility assay, while the latter more readily allowed quantification of IVM resistance levels. This highlights an issue which exists among the current suite of phenotypic assays, namely, that a single assay may not be the most suitable for resistance diagnosis for all drugs and helminth species (for example, [39]). Importantly though, the real-time nature of the RTCA readout in Figure 4D does allow for discrimination in the responses to IVM, however the variability seen in the data at these time points would suggest that such an assay would require a deal of careful standardisation before it could adequately quantify IVM resistance levels.

Recent programs to screen large libraries consisting of thousands of currently available drugs and other compounds have shown some promise for identifying new anthelmintics. For example, Abdulla *et al*. screened more than 2000 compounds *in vitro* against *S. mansoni* schistosomula and then progressed to screening 105 initial hits against adult stage parasites [8]. They used 200–300 schistosomula and 4–8 adult pairs per replicate and numerous additional screens when different time points were required. While robust data were generated, the program required a large scale effort. Even ignoring the time, effort and animal work required to produce the large number of worms, the screening alone took two full time researchers one month of training to identify phenotypes, three months to complete the primary screen with schistosomula and another month to screen the adult parasites. This laboratory and industry-based groups are developing automated video motility monitoring to improve scalability [41]. Initially developed for monitoring *C. elegans* sinusoidal movement the technique is now being adapted for parasites [42]. Currently, these systems require extensive mathematical modelling in the analysis programming that has to be customised by experienced personnel to each parasite and life cycle stage. While promising, this limits the applicability for the use of video monitoring for lab scale testing and development at this time. Microfluidic chips have also recently been developed and are showing great promise for screening of *C. elegans*. With innovative micro-channels to direct worms and micro-suction valves that trap individual worms, this device can sort whole worms depending on phenotype [43], [44]. This live, whole worm sorting is combined with florescence and digital imaging and permits phenotypic screening down to sub-cellular resolution. The limitations are that the microfluidic chambers are limited by size and adult parasites of many species are too large to be screened. While currently behaviour and neural function of *C. elegans* have been the focus of microfluidics research, it is feasible that these units could be adapted to monitor drug effects on larval parasites [45]. As with video-based monitoring,all these new technologies will have a place with the RTCA unit at various stages of the drug screening and resistance detection pipeline in the future.

As previously described, the E-plates contain 96 wells in a standard microtiter plate format, with up to 96 wells being monitored at any one time. The ease of experimentation enables the simultaneous monitoring of different species or developmental stages on the same plate. The RTCA unit that we used was the original single plate xCELLigence model (RTCA SP instrument). However, Roche Inc. recently released a multi-plate unit that can monitor up to six plates (576 samples) simultaneously. Additionally, a soon to be released 384 well model will assist scale up of larval assays, allowing for testing of additional samples with fewer larvae per well. These larger scale applications could be adapted to incorporate robotic handling for use with helminth eggs or larvae to streamline the scale-up in drug discovery programs. Post-genomic methods to determine the function of parasite genes and proteins are being developed [46], [47], and in time this will result in a suite of druggable targets. However, the lack of a high throughput objective tests for anthelmintic effectiveness represents a significant bottleneck that hampers the exploitation of this new post-genomic information [1], [8], [11], [22]–[24], [26]. Other xCELLigence models, such as the RTCA dual plate unit, are small, portable and powered by a laptop computer via USB connection. Such units may enable assessment of anthelmintic activity in field settings where drug efficacy studies are undertaken.

The high sensitivity of this motility assay allows for detection of subtle differences following drug application with relative ease. Subtle drug effects are often overlooked when existing methods are used. For example, the effect of low PZQ concentrations on schistosomes we observe has until now gone unnoticed (Figure 4A). The ability to measure parasite motility with enhanced sensitivity in a user-friendly manner will prove valuable in the detection of emerging drug resistance, a rapidly growing area of concern for human helminth infections [48]–[50], thereby facilitating early intervention.

A unique aspect to RTCA for monitoring helminth motility is its ability to continuously assess movement in real time. While the full analysis requires conversion of raw data into a motility index, effects on parasite motility can be easily monitored as the experiment progresses (Figure 1). Moreover, live data can be simply exported for motility index analysis during the experimentation period. This is particularly useful for experimental design using adult stage worms which are less amenable to long-term culture than are larval stages. The ability to measure motility (and set baseline parameters) prior to addition of drugs ensures that adequate replicates of healthy motile worms are recorded for each treatment condition, a consideration that assists data interpretation and statistical power.

The added benefit of real time, intervention-free monitoring is that IC_50_ values can be generated for any number of time points within a single sample. Firstly, this allows fewer parasites to be used with less set-up time required. Secondly, this enables greater insight into defining the optimal time points for the detection of resistance (for example, Fig 4C) and timing of treatment. Thirdly, combination treatments can be more easily analysed, either with concurrent or successive applications. The real time nature of the assay allows multiple factors that affect resistance to be assessed, such as the kinetics of LEVA resistance [51]–[53], or early and/or late effects that may be overlooked when defined time points are recorded. For example, when we cultured schistosomes in 50 and 100 ng/ml PZQ (Figure 2A), there was an immediate effect on motility upon addition of drug, followed by a gradual recovery of motility from approximately 15–72 hours. A second example is the difference between the IC_50_ values of LEVA- and IVM-resistant *Haemonchus* L_3_ over time (Figures 4C and 4D), where significant differences in motility were detected between resistant and sensitive lines until 12 hours following addition of both drugs. Thereafter the difference in motility between resistant and susceptible parasites was maintained for LEVA resistant L_3_. In contrast, IVM-treated parasites showed similar motility between resistant and sensitive lines after 24 hours. Many anthelmintic drugs are metabolised within hours, so this data will be critical in designing treatment programs to maximise drug effectiveness and reduce costs. One drawback of monitoring slow acting drugs with this technique, such as IVM and TBZ (Figures 3B and 4D), is that the IC_50_ 95% confidence intervals can be substantial in the early period of the experiment. The reasons for this are unclear but we suspect that it reflects the slow induction of paralysis, hence the increased variability between samples.

The versatility of this RTCA technique for measuring motility of microorganisms may result in a wide range of applications. It could be used to assess the effects on helminths of treatments other than drugs, including antibodies and other immune interventions, or gene silencing approaches where the phenotype affects motility [54], [55]. Modification of the RTCA method for use with a range of other difficult to assess organisms is feasible. The free-living nematode *Caenorhabditis elegans* is widely used as a model for parasitic nematodes due to its functional and biotechnological tractability [56]–[59]. Adult *C. elegans* range from 1–2 mm in length, so it is likely that their motility in liquid culture could be easily measured using a modified RTCA approach [57]–[62]. The range of potential species that may be monitored with this technique is extensive, including agricultural, medical and veterinary pests and pathogens such as ticks, fleas, aphids, mites and dipteran larvae [63]–[65].

In conclusion, we present a novel use of a Real Time Cell Assay device (xCELLigence) that can simply and objectively assess the effectiveness of anthelmintic drugs in real time by measuring motility in a high-throughput, reproducible fashion with minimal effort and training required. While originally designed for real time measurement of cell growth, the device is amenable to high throughput screening of a range of developmental stages of different human and livestock helminth parasites. This method is envisaged to be applicable for the majority of helminth species and developmental stages where egg hatch assays or motility is accepted as a measure of worm viability. We predict that the method could be applied to other large pathogens or pests that can survive and be motile in liquid culture in a 96 well plate (or smaller). Moreover, new models of the xCELLigence are soon to be released by Roche Inc, displaying improved sensitivity and increased scale-up potential. The widespread use of this device to screen for new therapeutics or emerging drug resistance will be an invaluable asset in the fight against the wide range of biomedical and veterinary helminths that plague our planet.
